# Is Irritable Bowel Syndrome Considered in Clinical Trials on Physical Therapy Applied to Patients with Temporo-Mandibular Disorders? A Scoping Review

**DOI:** 10.3390/ijerph17228533

**Published:** 2020-11-17

**Authors:** Daiana P. Rodrigues-de-Souza, Javier Paz-Vega, César Fernández-de-las-Peñas, Joshua A. Cleland, Francisco Alburquerque-Sendín

**Affiliations:** 1 Department of Nursing, Pharmacology and Physical Therapy, Faculty of Medicine and Nursing, University of Córdoba, 14004 Córdoba, Spain; drodrigues@uco.es (D.P.R.-d.-S.); jespartano16@gmail.com (J.P.-V.); falburquerque@uco.es (F.A.-S.); 2 Department of Physical Therapy, Occupational Therapy, Rehabilitation and Physical Medicine, Universidad Rey Juan Carlos, 28922 Alcorcón, Spain; 3 Cátedra Institucional en Docencia, Clínica e Investigación en Fisioterapia: Terapia Manual, Punción Seca y Ejercicio Terapéutico, Universidad Rey Juan Carlos, 28922 Madrid, Spain; 4 Doctor of Physical Therapy Program, Department of Public Health and Community Medicine, Tufts University School of Medicine, Boston, MA 02155, USA; joshua.cleland@tufts.edu; 5 Maimonides Biomedical Research Institute of Cordoba (IMIBIC), 14004 Córdoba, Spain

**Keywords:** temporomandibular pain, irritable bowel syndrome, eligibility, clinical trial, physical therapy

## Abstract

The aim of the current scoping review was to identify if the presence of irritable bowel syndrome was included as eligibility criteria of participants included in clinical trials investigating the effects of physical therapy in individuals with temporomandibular pain disorders (TMDs). A systematic electronic literature search in the Web of Science database was conducted. Scientifically relevant, randomized clinical trials (those cited in other studies at least 5 times, or clinical trials published in high-impact journals, i.e., first and second quartiles (Q1-Q2) of any category of the Journal Citation Report (JCR)) evaluating the effects of any physical therapy intervention in patients with TMDs were included. The Physiotherapy Evidence Database (PEDro) scale was used to evaluate the methodological quality of the selected trials. Authors affiliated to a clinical or non-clinical institution, total number of citations, objective, sex/gender, age, and eligibility criteria in each article were extracted and analyzed independently by two authors. From a total of 98 identified articles, 12 and 19 clinical trials were included according to the journal citation criterion or JCR criterion, respectively. After removing duplicates, a total of 23 trials were included. The PEDro score ranged from 4 to 8 (mean: 6.26, SD: 1.48). Based on the eligibility criteria of the trials systematically reviewed, none considered the presence of comorbid irritable bowel syndrome in patients with TMDs. The comorbidity between TMDs and irritable bowel syndrome is not considered within the eligibility criteria of participants in highly cited clinical trials, or published in a high-impact journal, investigating the effects of physical therapy in TMDs.

## 1. Introduction

Temporomandibular disorders (TMDs) consist of a group of conditions affecting the temporo-mandibular joint and/or masticatory muscles that influence the orofacial region and represent one of the main causes of non-dental facial pain [1].

Pain, restricted range of motion, and crepitation (clicking sound of the temporo-mandibular joint) are symptoms commonly experienced by the patients [2]. In addition, TMDs can also be associated with the presence of sleep bruxism (clenching and grinding of teeth during sleep), dental malocclusion [3], and mood disorders (e.g., anxiety or depression) [4]. The prevalence of TMDs ranges from 5% to 12% in the general population [5] with a higher proportion of women being afflicted [6]. The etiology of TMD is not completely understood, and it appears to be multifactorial.

Patients with TMDs usually exhibit comorbidities with other pain conditions, such as primary headaches [7,8], fibromyalgia [9], or chronic fatigue syndrome [10]. In fact, the presence of these comorbid conditions is associated with higher levels of hyperalgesia [11] and an increase in the intensity and duration of TMD symptoms [12]. Additionally, TMD is not only concomitant with musculoskeletal pain conditions, but also with other systemic diseases [13]. Of particular interest is the association between irritable bowel syndrome and TMD. In fact, it has been reported that 64% of individuals with TMDs also suffer from irritable bowel syndrome [14,15]. Both conditions share common risk factors. For instance, anxiety causes visceral hypersensitivity and generates visceral excitability [14], but it is also considered a potential risk factor for the development of TMD [16,17]. Indeed, the development and maintenance of visceral hypersensitivity in animal studies, after applying a distal somatic lesion and stress, mimics some features of TMD and comorbid irritable bowel syndrome [18]. Additionally, patients with irritable bowel syndrome and/or TMD suffer from additional (widespread) pain not related to the primary complaint resulting in overlapping of pain syndromes, making them difficult to assess and manage [19,20].

The potential relationship between these two entities requires a clear definition of the participants recruited in clinical trials investigating the effects of different treatment modalities for patients with a primary complaint of TMDs. Identifying which individuals could exhibit comorbid conditions such as irritable bowel syndrome, which might not be specifically treated with the proposed intervention, is essential. Among those potential therapeutic approaches proposed for the management of individuals with TMD, physical therapy has shown to be beneficial for these patients [21,22]. Meta-analyses represent the highest level (1a) of evidence for determining the effects of an intervention as they are based on randomized clinical trials. Despite the increases in the number and quality of clinical trials including patients with TMD, the influence of relationships among people with TMDs with psychological and somatic-visceral conditions could lead to uncertainty regarding the etiology of symptoms and may influence the clinical outcomes. If comorbid conditions are ignored in the selection criteria of clinical trials, this may limit the internal validity and lead to loss of potential benefits of physical therapy interventions, which mainly target the musculoskeletal, but not the visceral, system [23]. It has recently been observed that 80% of relevant clinical trials investing the effects of physical therapy in people with neck pain did not consider a potential visceral cause of neck pain symptoms [24]. No study has previously investigated this topic in clinical trials including individuals with TMDs.

Therefore, the objective of this scoping review was to identify whether the presence of irritable bowel syndrome was assessed within the eligibility criteria of participants included in clinical trials investigating the effects of physical therapy interventions in individuals with TMDs.

## 2. Methods

A scoping review following the methodological framework suggested by Arksey and O’Malley [25] was conducted: 1, identify a research question; 2, identify relevant studies; 3, study selection; 4, chart the data; 5, collate, summarize, and report the results. This scoping review was conducted following The Preferred Reporting Items for Systematic Reviews and Meta-Analyses Extension for Scoping Reviews (PRISMA-ScR) to ensure a transparent and accurate reporting structure [26]. This scoping review was prospectively registered on the Open Science Framework Registry (https://doi.org/10.17605/OSF.IO/GB7DA).

### 2.1. Identify the Research Question

The key research question of this scoping review was as follows: Is the presence of comorbid visceral disorders, i.e., irritable bowel syndrome, considered in the eligibility criteria in scientifically relevant clinical trials investigating the effects of physical therapy interventions in individuals with TMDs?

### 2.2. Identify Relevant Studies

A systematic electronic literature search in the Web of Science (WOS) database was conducted from the inception of the database to 10th September 2020. The reference lists of papers identified in the databases were also searched. Common journals within the topic of TMD were also hand-searched to identify articles that might have been missed in the database and reference list searches. The database literature search was conducted by two different researchers with the assistance of an experienced health science librarian. Searches were limited to human studies, clinical trials, and without language restrictions. The following terms were combined by using Boolean operators: “temporomandibular disorders” OR “temporomandibular pain”, AND “physical therapy” OR “physiotherapy”.

### 2.3. Study Selection

This review used the PCC mnemonic (Population, Concept, and Context) to define the inclusion criteria [27].

Population: Adults diagnosed with TMDs according to the Research Diagnostic Criteria for TMD (RDC/TMD) [28] or the Diagnostic Criteria for TMD (DC/TMD) [2].

Concept: Randomized clinical trials evaluating any physical therapy intervention, alone or combined with, in individuals with TMD. Updated studies, systematic or narrative reviews, and meta-analyses, or any other design different than clinical trial, were excluded.

Context: Articles were considered of scientific relevance if they fulfilled the following: 1, clinical trials cited in other studies at least 5 times (citation criterion); or, 2, clinical trials published in high-impact journals, i.e., first or second quartiles (Q1-Q2) of any category of the Journal Citation Reports (JCR) assessed in the year of publication of the study, according to the WOS (JCR-impact factor criterion).

The study selection was performed independently by two researchers by reviewing the title and abstract of the text. A full text read of potential eligible trials was conducted.

In case of disagreement, a third researcher decided the final inclusion or exclusion of the study. All data were saved and managed with Microsoft Office^®^ (Microsoft^®^, Washington, USA).

### 2.4. Chart Data

Data extraction was conducted using a “data charting form” in which a descriptive summary of the results is generated [25]. A data charting form was developed for this scoping review to identify the variables that correspond with the research question. Data were extracted independently by two authors using a data charting form including authors (grouped by clinicians, i.e., affiliated to a clinical entity, or academics, affiliated to a non-clinical institution), the Physiotherapy Evidence Database (PEDro) score, the total number of citations in WOS, the objectives, sex/gender, age, and eligibility (inclusion/exclusion) criteria to select the participants to be included from each study. Two researchers completed the chart data and had to achieve a consensus on each item. If disagreement occurred, a third researcher participated in the decision to reach resolution.

### 2.5. Methodological Quality

The Physiotherapy Evidence Database (PEDro) scale was used to evaluate the methodological quality of the selected studies. This scale consists of 10 items scored as yes (0) or no (1) according to whether the criterion is clearly satisfied. A score ≥ 5 points was considered of high methodological quality. Scores were extracted from the PEDro database if available. If a trial was not assessed by PEDro, the scale was applied by two researchers following current guidelines [29]. The PEDro scale has been shown to exhibit adequate validity and reliability and is a common used tool to determine methodological quality of clinical trials [30].

## 3. Results

### 3.1. Study Selection

From a total of the 98 identified articles, 67 studies were excluded for not meeting the eligibility criteria, and one [31] because it was a duplication of another trial [32] with the same authors and similar study characteristics. One study was excluded because it did not use RDC/TMD or DC/TMD [33]. Finally, 12 clinical trials were included according to the citation criterion, and another 19 according to the JCR criterion. Eight of the articles fulfilled both criteria; therefore, the duplicates were removed.

**Figure 1:**
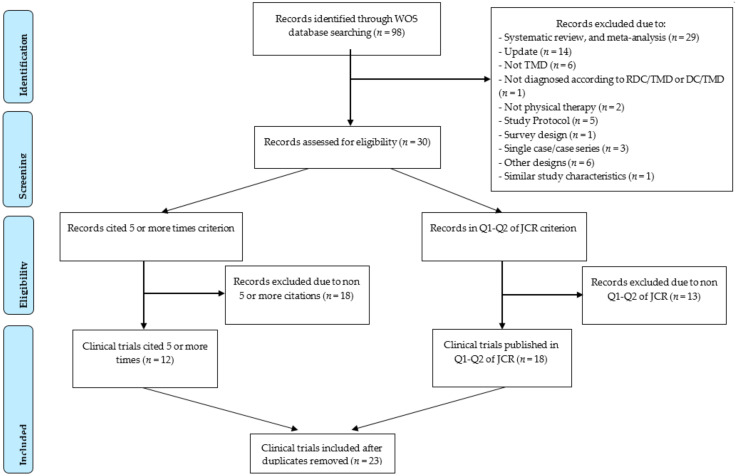
PRISMA Extension for Scoping Reviews (PRISMA-ScR) flow diagram.

Finally, a total of 23 trials were included in the literature data mapping [32,34,35,36,37,38,39,40,41,42,43,44,45,46,47,48,49,50,51,52,53,54,55]. Figure 1 shows the flow chart of the study selection.

### 3.2. Study Characteristics

Data extracted from the most cited trials, trials published in Q1-Q2 of any category of the JCR, and those fulfilling both criteria are described in Table 1, Table 2 and Table 3. The total sample size of the trials was 1175 participants (82.8% women) with TMD. A greater number of women participated in all trials, with only one exception [53]. Six trials only recruited women [39,44,45,47,50,52]. The mean age of the participants ranged between 24 and 50 years (mean: 34.9, SD: 7.4 years).

### 3.3. Methodological Quality

The methodological quality score ranged from 4 to 8 (mean: 6.25, SD: 1.5) out of a maximum of 10 points. All clinical trials were cited 5 or more times (mean: 6.2, SD: 1.3), and 15 out of 18 published in Q1-Q2 of JCR (mean: 6.8, SD: 1.4) were scored with 5 points or more; therefore, they were considered of high methodological quality. Only three trials were considered of low methodological quality since they had a PEDro score of 4 points [41,42,44].

### 3.4. Inclusion Criteria in Clinical Trials

To simplify, the results of both inclusion and exclusion criteria are presented separately. After examining inclusion criteria, no clinical trial considered participants with possible visceral pain. In general, the inclusion criteria were “Patients with TMDs” or “female and male volunteers with TMD” or “Myogenic pain and continuous pain report of more than 3 months”, with little detailed information. In four clinical trials, in addition to TMD, other conditions such neck pain [47], tinnitus [43,53], and restricted mobility of the upper cervical spine [34] were also inclusion criteria.

### 3.5. Exclusion Criteria in Clinical Trials

No trial specified any visceral disorder as an exclusion criterion. The exclusion criteria were highly diverse including degenerative/collagen disease of the TMJ [42], systemic diseases [32,34,39,44,48], autoimmune diseases [41], cardiovascular diseases [38], systemic inflammatory disorders [34,37,38,45,48], tumors [34,43,53], whiplash [34,47], neuromuscular diseases [47], vestibular alterations [40], bleeding disorders [42], pregnant women [45,52], mental [46] or psychiatric [36,38,39,42,45] disorders, headaches [42,51], fibromyalgia [39,43,45], acute infections [47,51], or neurological diseases [32,36,37,40,42,43,45,48,53,55].

Additionally, the use of medication potentially affecting pain perception (e.g., anti-inflammatories [34,37,41,43,48,50,52] or muscular relaxants [41,48,50,52]) were also specified as exclusion criteria in some studies.

## 4. Discussion

### 4.1. Eligibility Criteria Used in Clinical Trials to Select Patients with TMD

This scoping review aimed to identify if the potential comorbidity between TMD and visceral pain, particularly irritable bowel syndrome, is considered in the eligibility criteria of highly cited clinical trials, or published in high-impact journals, investigating the effects of physical therapy treatment in TMD patients. The authors did not find consideration of this comorbidity, not only between TMDs and irritable bowel syndrome, but also with any visceral pain condition that could influence TMDs.

All trials included individuals with TMD diagnosed according to the RDC/TMD criteria [28], except Espi-López et al. [48] and Nagata et al. [46] who used the DC/TMD criteria [2] as the main inclusion criterion. Further, some trials specified the reproducibility of symptoms during physical examination [48,55], jaw movement [42], and a minimum pain intensity of 3–4 points [37,45], among others inclusion criteria. All these symptoms mainly refer to increased local sensitization and are focused on musculoskeletal impairments, ignoring a potential visceral issue. Among the exclusion criteria, most referred to trauma, surgery, previous treatments, or systemic medical musculoskeletal conditions, but without reference to any visceral condition. Current findings suggest that despite the progress of research and treatment for TMDs, visceral conditions, which are highly prevalent, are potentially important to address when assessing the effects of treatments targeting the musculoskeletal system, such as physical therapy, in individuals with TMD.

### 4.2. Comorbidity between Irritable Bowel Syndrome and TMD

The comorbidity between TMD and other clinical conditions, such as irritable bowel syndrome, chronic fatigue syndrome, fibromyalgia, interstitial cystitis, post-concussive syndrome, chemical sensitivities, and chronic pelvic pain, was described twenty years ago [56]. Nevertheless, the complexity of the nature and consequences on TMDs, as well as the necessity of developing new strategies to effectively diagnose, prevent, and treat these chronic and debilitating comorbid conditions, was described just ten years ago [57]. In fact, TMD does not often occur in isolation. For instance, TMD could be a manifestation of symptoms associated with widespread syndromes such as fibromyalgia, irritable bowel syndrome, or whiplash-associated disorders [57,58]. However, TMD could also be a promoting factor for more widespread pain symptoms. In fact, Pfau et al. identified two groups of patients with TMD, sensitive and non-sensitive, based on the presence of altered nociceptive processing and comorbid fibromyalgia [59]. This bidirectional relationship potentially increases the relevance to clinically identify comorbid syndromes for patients with TMD.

Although determining the mechanisms underlying the comorbidity between these conditions is beyond the scope of the current review, the topic of central sensitization will be discussed due to its potential application for clinical practice. It seems that enhanced pain perception, altered brain activation, dysregulation in immune and neuroendocrine function, and genetic susceptibility are common mechanisms in several pain syndromes, called central sensitivity syndromes [60]. Thus, altered pain perception has been well described in patients with TMD [61,62,63], or with irritable bowel syndrome [64,65] separately, and when both conditions are present, more hypersensitivity is also present [60]. This altered nociceptive pain system has a common anatomic substrate since a reduction in the gray matter within the limbic system and insula has been found in patients with TMD [66] or with irritable bowel syndrome [67]. Similarly, TMD and irritable bowel syndrome share psychosocial distress as common associated risk factors, suggesting that comorbid clinical entities could encompass a continuum of syndromes with common risk factors [68].

### 4.3. Clinical Relevance of Current Findings

The presence of comorbid conditions of different origin (e.g., musculoskeletal vs. visceral) should be considered in clinical practice. For instance, altered pain perception in terms of sensitization processes has been used to classify patients with TMD, which can help to differentiate therapeutic strategies [69]. Similarly, since visceral pain enhances central sensitization [70], assessment and treatment of visceral pain comorbidities should be integrated as part of TMD management. In fact, exacerbation of pain symptoms when two conditions exist is labeled as functional somatic syndrome [62], which should be considered in the management of chronic pain conditions.

Early recognition of comorbid syndromes of different etiology, but common pain mechanisms, may identify subgroups of patients with different etiologies and needs of treatment [71]. Comorbid pain conditions should not be ignored when a physical therapy treatment is tested, which potentially occurred in the identified trials. This is highly relevant considering that visceral pain shares several features with pain from musculoskeletal deep somatic structures but clearly require different therapeutic strategies [16]. Future studies should investigate the effects of multimodal therapeutic programs considering the presence of visceral comorbidities (e.g., irritable bowel syndrome) in those individuals with a primary musculoskeletal complaint (e.g., TMD). Further, the identification of comorbid syndromes in people participating in physical therapy clinical trials could also increase the internal and external validity of the designs, leading to potentially different clinical outcomes.

### 4.4. Strengths and Limitations

The results from this scoping review should be analyzed according to its potential strengths and limitations. Strengths include a comprehensive literature search, methodological data extraction, and the inclusion of highly cited clinical trials, and/or published in high-impact journals, investigating physical therapy for the management of TMDs. Among the limitations, only 23 trials achieved the level of quality required in this scoping review. This number could be considered relatively small; however, the direction of the results was homogeneous, and the inclusion of a greater number would likely not alter our findings. In addition, physical therapy interventions were heterogeneous ranging from manual therapy to exercise alone or combined with surgical procedures. Second, the search was only conducted in a single database, WOS, which is the only one offering the index classification by JCR. It would be necessary to extend the search to a greater number of databases for confirming these results. Finally, 80% of the patients with TMD included in the clinical trials were female, which may be related to the fact that TMD is more prevalent in females than in males and also that a greater frequency of comorbidity in pain syndromes and TMDs is present in females [68]. Since women exhibit greater susceptibility of developing central sensitivity syndromes [72], future clinical trials investigating the effects of physical therapy interventions for the management of TMDs should consider potential gender differences in their eligibility criteria.

## 5. Conclusions

This scoping review found that highly cited clinical trials, or published in high-impact journals, investigating the effects of physical therapy interventions in individuals presenting with TMD lacked the consideration of comorbid irritable bowel syndrome into their eligibility criteria. These results suggest the influence of visceral pain in TMDs may potentially be underestimated. Since comorbid visceral pain, particularly irritable bowel syndrome, promotes TMD symptoms, ignoring its influence may result in an inaccurate estimation of usefulness of musculoskeletal pain interventions in this population. Therefore, more stringent inclusion and exclusion criteria may be required in clinical trials including participants with TMDs.

## Figures and Tables

**Table 1:** Characteristics of clinical trials including patients with temporomandibular disorders according to the citation criteria. F—Female; M—Male; TMD—Temporomandibular disorders; TMJ—Temporomandibular joint; RDC/TMD—Research Diagnostic Criteria for Temporomandibular disordes; VAS—visual analoge scale.

Study	Clinical/Non-Clinical	Number of Cites	PEDRO Score	Objective	Participants (Gender)	Mean Age	Inclusion Criteria	Exclusion Criteria
Amaral et al., 2013	Clinical: 0; Non-clinical: 6	6	6/10	To analyze the immediate effect of non-specific mandibular mobilization on postural control in patients diagnosed with TMD in two visual conditions: eyes open and eyes closed.	N = 50 36 F/14 M	27 years	Age between 20 and 40 years with full permanent dentition; the TMD group had TMD, mandibular deviation, or deflection.	Crossbite, open bite, or overbite; prognathism or retrognathism; dental prosthesis; undergoing orthodontic treatment or physical therapy; neurological and/or orthopedic disorders affecting body balance; using orthopedic insoles; having low blood pressure (BP); auditory and/or vestibular alterations; using medication for balance; younger than 20 years, older than 40 years; overweight.
							RDC/TMD sub-type: Non specified	
Cuccia et al., 2010	Clinical: 0; Non-clinical: 4	25	6/10	To test the effect of osteopathic manual therapy in patients with TMD	N = 50 28 F/22 M	40 years	Patients with TMD; with a temporomandibular index reference value of ≥0.08 ± 0.10; pain intensity of at least 40 mm on a visual analogue scale (VAS).	History of adverse effects with osteopathic treatment; being under orthodontic treatment or under treatment for TMD; previous treatment; regular use of analgesic or anti-inflammatory drugs; dental prosthesis; presence of other oro-facial condition; neurological or psychiatric disorders and systemic inflammatory disorder.
							RDC/TMD sub-type: Non specified	
Gomes et al., 2012	Clinical: 0; Non-clinical: 4	12	5/10	To evaluate the effect of cathodal high-voltage electrical stimulation on pain in women with TMD	N = 25 All F	24 years	Women with pain in TMJ and/or masticatory muscles, pain and/or fatigue in the masticatory muscles during functional activities for a at least one year and a maximum of five years. RDC/TMD sub-type: Non specified	Undergoing orthodontic treatment; drug therapy (pain relievers, anti-inflammatories, muscle relaxants); physical therapy treatment
Nascimento et al., 2013	Clinical: 2; Non-clinical: 4	7	6/10	To evaluate the effects of physical therapy and anesthetic blockage of the auriculotemporal nerve for TMD.	N = 20 All F	41 years	Adults with disc displacement and arthralgia with pain intensity from 3 to 9 on a visual analogue scale (VAS)	Previous treatment with pharmacotherapy; use of occlusal appliances; symptoms related to disease in other parts of the stomatognathic system (e.g., toothache, neuralgia); systemic disease (e.g., rheumatoid arthritis); fibromyalgia and history of psychiatric disorders.
							RDC/TMD sub-type: arthralgia—Axis I Groups IIA, IIB, and IIIA.	
Tuncer et al., 2013	Clinical: 0; Non-clinical: 4	35	7/10	To determine the effectiveness of home physical therapy (HPT) alone and manual therapy (MT) on pain intensity and pain-free maximum mouth opening in patients with TMD.	N = 40 31 W/9 M	36 years	Adults with myogenous TMD. Pain on palpation of at least three of 12 muscular points bilaterally. Adults with diagnosis of anterior disc displacement with reduction, and painful clicking, crepitation or pain on opening and loaded closing with reproducibility in at least 2 of 3 consecutive trials RDC/TMD sub-types: myogenous TMD—Axis I Groups IA and IB arthralgia-anterior disc displacement with reduction—Axis I group IIA	Disc displacement without reduction; arthritis or TMJ—categories IIb and III of the RDC/TMD; previous surgery related; TMD treatment within the previous three months; neurological or psychiatric disorders that could interfere with the procedure and intake of any medication that affects the musculoskeletal system.

**Table 2:** Characteristics of clinical trials including patients with temporomandibular disorders according to the JCR criteria. F—Female; M—Male; MRI—Magnetic Resonance Imaging; TMD—Temporomandibular disorders; TMJ—Temporomandibular joint; RDC/TMD—Research Diagnostic Criteria for Temporomandibular disordes; DC/TMD—Diagnostic Criteria for Temporomandibular disordes.

Study	Clinical/ Non-Clinical	Number of Cites	PEDRO Score	Objective	Participants (Gender)	Mean Age	Inclusion Criteria	Exclusion Criteria
Bas et al., 2018	Clinical: 0; Non-clinical: 4	1	4/10	To evaluate the effect of exercise and massage on range of movement and pain after arthrocentesis therapy in patients with TMJ disc displacement without reduction.	N = 27 25 F/ 2 M	33 years	Diagnosed as TMJ disc displacement without reduction, failed conservative, non-surgical treatment for a minimum of 2 months. MRI study done for the assessment of TMJ internal derangement.	Presence of known connective tissue or autoimmune diseases; previous surgery; degenerative joint disease; history of major jaw trauma; concurrent use of steroids, muscle relaxants, or narcotics.
							RDC/TMD sub-type: arthralgia—Axis I Groups IIA, IIB, and IIIA.	
Calixtre et al., 2019	Clinical: 0; Non-clinical: 6	5	8/10	To determine whether mobilization of the upper cervical region and cranio-cervical flexor training can improve orofacial pain in women with TMD when compared to a control group.	N = 61 All F	26 years	Female; aged between 18 and 40 years old; orofacial pain for at least 3 months; baseline pain score ≥3 points on a NPRS; and diagnosis of myofascial or arthrogenic TMD	Pregnancy; diagnosis of fibromyalgia, rheumatic or neurologic issues; history of neck or jaw fracture; dental loss (except for third molars, when extracted more than 6 months ago); and previous orofacial treatment (such as orthodontics or physiotherapy in the previous 6 months).
							RDC/TMD sub-type: arthralgia—Axis I Groups IA, IB, IIA, IIB, IIIA, and IIIC	
Capan et al., 2017	Clinical: 0; Non-clinical: 8	2	4/10	To investigate the impact of a comprehensive and multicomponent early supervised rehabilitation program in comparison with homebased exercise after TMJ condylar discopexy	N = 31 30 F / 1 M	32 years	Clinical diagnosis of TMD dis displacement without reduction (history of reduction in mandibular opening >6 months, unassisted mandibular opening ≤35 mm, TMJ pain (VAS >5 cm), deflection of the mandibular opening pathway to the ipsilateral side; restrictions in lateral movements of the ipsilateral side, no longer present joint sounds); MRI diagnosis of disc displacement without reduction; RDC/TMD sub-type: arthralgia—Axis I Groups IIA, IIB, and IIIA.	Presence of other disorders involving the TMJ (e.g., degenerative joint disease or collagen vascular disease); history of major jaw trauma; dentofacial deformity; psychiatric illness; chronic headache; inflammatory disorders; bleeding disorders; neurological disorders
Delgado de la Serna et al., 2020	Clinical: 2; Non-clinical: 4	1	8/10	To evaluate the effectiveness of adding specific cervico-mandibular manual therapies into an exercise and educational program on clinical outcomes in people with tinnitus associated with TMD.	N = 61 36 F / 25 M	49 years	Age 18–65 years; diagnosis of tinnitus attributed to TMD diagnosed according to the RDC/TMD criteria	Diagnosis of ear, nose, and throat medical pathology underlying the tinnitus; neurological disorders; inability to read, understand, and complete the questionnaires or understand and follow commands (e.g., illiteracy, dementia, or blindness); comorbid fibromyalgia syndrome; had received physiotherapy or other treatment; contraindication to physical therapy as noted in the patient’s Medical Screening Questionnaire (i.e., tumor, fracture, rheumatoid arthritis, osteoporosis, history of steroid use).
							RDC/TMD sub-type: Non specified	
Espí-López et al, 2020	Clinical: 0; Non-clinical: 6	0	7/10	To evaluate whether a combined program of manual therapy techniques plus traditional splint therapy improves pain and clinical dysfunction in subjects with TMD	N = 16 13 F / 3 M	30 years	Aged 18 to 65; diagnosed with mild TMD signs and symptoms according to Helkimo Index and diagnosed with myofascial TMD according to DC/TMD	Systemic, rheumatic, or central nervous system diseases; surgical history in TMD area; previous physical therapy treatments; diagnosed with other orofacial or TMJ disk disorders; vertebral artery compromise test; cerebrovascular disorders; use of analgesics or muscle relaxants; use of splint 1 month before the start of the study.
							DC/TMD sub-type: Myofascial	
Giro et al., 2016	Clinical: 0; Non- clinical: 8	1	4/10	To evaluate the impact of treatment with instruction about TMD or education associated with self-care therapies on the mandibular movement pattern in women with TMDs.	N = 42 All F	36 years	Between 18 and 50 years of age; diagnosis of TMD according to RDC-TMD criteria; presence of pain for more than 3 months; pain intensity higher than 3 points on a NPRS; had received no treatment or insufficient treatment for this painful condition and had not started any treatment for other painful conditions; and manifested presence of natural dentition or fixed prostheses with posterior occlusal stability.	Severe malocclusions; debilitating systemic diseases; presence of a cardiac pacemaker (to avoid possible interference with the kinesiograph).
							RDC/TMD sub-type: Axis I Groups IB, IIA, and IIB	

**Table 3:** Characteristics of clinical trials including patients with temporomandibular disorders fulfilling both citation and JCR criteria. F—Female; M—Male; TMD—Temporomandibular disorders; TMJ—Temporomandibular joint; RDC/TMD—Research Diagnostic Criteria for Temporomandibular disordes; VAS—Visual Analogue Scale.

Study	Clinical/Non-Clinical	Number of Cites	PEDRO Score	Objective	Participants (Gender)	Mean Age	Inclusion Criteria	Exclusion Criteria
Craane et al., 2012	Clinical: 0; Non-clinical: 4	27	8/10	To investigate the effect of physical therapy on pain and mandibular function in patients with anterior disc displacement without reduction (ADD-R) of the TMJ.	N = 49 47 F/2 M	36 years	Strictly satisfied the RDC-TMD criteria for disc displacement without reduction and pain experienced during the first examination of ≥ 35 mm on a VAS. RDC/TMD sub-type: arthralgia—Axis I Groups IIB and IIC.	Orofacial trauma; systemic disorders; cervical disorders; neurologic disorders; drug or alcohol abuse; use of antidepressants or hormonal medication; not receiving therapy for symptoms of TMD within the preceding 2 months.
Dworkin et al., 2002	Clinical: 0; Non-clinical: 8	146	5/10	To compare usual conservative treatment of TMD by clinical TMD specialists with a structured self-care intervention, targeted to clinic cases independent of TMD physical diagnosis, who were reporting minimal levels of psychosocial dysfunction	N = 124 109 F/21 M	37 years	Self-report of facial ache or pain in the muscles of mastication, the TMJ, or the region in front of the ear or inside the ear; report of stiffness or other symptoms of discomfort in the orofacial region; age between 18 and 70 years.	Pain attributable to confirmed migraine or head pain condition other than tension headache; acute infection or other significant disease of the teeth, ears, eyes, nose, or throat; debilitating physical or mental illness; necessity for emergency TMD treatment; inability to speak or write English.
							RDC/TMD sub-type: Non specified	
Haketa et al., 2010	Clinical: 0; Non-clinical: 5	37	5/10	To evaluate the therapeutic efficacy between two treatment options for anterior disc displacement without reduction: one an occlusal splint, and the other joint mobilization self-exercise.	N = 52 46 F/6 M	38 years	Adults with pain during mouth-opening on the TMJ affected side; over 2 weeks after the onset of anterior disc displacement without reduction; maximum mouth opening of less than 40 mm; MRI-confirmed anterior disc displacement without reduction.	Unwilling or unable to receive splint and/or exercise therapy; presence of systemic bone or joint disease; taking regular medication such as analgesics, anti-anxiety drugs, antidepressants, and psychotropics; missing teeth and/or having a removable denture, but having a fixed partial denture restoration over 1 year.
							RDC/TMD sub-type: arthralgia—Axis I Groups IIB and IIC.	
Michelotti et al., 2004	Clinical: 0; Non-clinical: 6	82	5/10	To compare the short-term efficacy of patient education only versus the combination of patient education and home exercises for the treatment of myofascial pain of the jaw muscles	N = 70 62 F/8 M	30 years	Pain recurrent or constant for more than 3 months; spontaneous pain in the last week of >30 on a VAS	Objective evidence of TMJ pathology or dysfunction; arthrogenous TMD; other orofacial pain conditions; other TMD treatments within the last 3 months; neurologic or psychiatric disorders; history of pain medication abuse or current abuse
							RDC/TMD sub-type: myofascial—Axis I Groups IA and IB	
Oliveira et al., 2015	Clinical: 0; Non-clinical: 7	15	8/10	To test whether transcranial direct current stimulation could influence the effects of exercises on participants with TMD and chronic pain.	N = 32 29 F/3 M	25 years	Diagnosed with TMD based on the RDC/TMD; pain intensity equal to or over 4/10 on a VAS during the last 6 months. RDC/TMD sub-type: myofascial—Axis I Groups IA and IB	Individuals who had received any type of physiotherapy treatment in the last month; presence of rheumatic or cardiovascular diseases or convulsion; presence of metal implant in the brain or skull.
Packer eta al., 2014	Clinical: 0; Non-clinical: 4	14	8/10	To evaluate the effects of upper thoracic manipulation on facial pain in women with TMD	N = 32 All F	25 years	Aged 18-40 years, diagnosis of myofascial TMD according to the RDC/TMD; pain or fatigue in the masticatory muscles from at least 6 months; diagnosis of neck pain based; BMI < 25 kg/m^2^.	Missing teeth (except third molars); use of complete or partial dentures; systemic neuromuscular disease; current treatment of TMD; red flag signal for malignant tumor, inflammatory disease, or infection that contraindicated the use of manual therapy; history of whiplash, surgery of the cervical spine, and having undergone spinal manipulation in the previous month; and diagnosis of arthralgia based on the RDC/TMD.
							RDC/TMD sub-type: myofascial—Axis I Groups IA and IB	
Yuasa and Kurita, 2001	Clinical: 0; Non-clinical: 2	139	5/10	To compare the effectiveness of nonsteroidal anti-inflammatory drugs (NSAIDs) and physical therapy for disk displacement without reduction with nontreatment controls.	N = 60 48 F/8 F	26 years	Unilaterally moderate or severe TMJ dysfunction lasting 2 weeks or more and MRI showing disk displacement without reduction and without osseous changes.	Pain other than in the TMJ region; myofascial pain dysfunction; undergone other treatment for the 4 weeks immediately before enrollment, as were patients who were unable to take NSAIDs.
							RDC/TMD sub-type: arthralgia—Axis I Groups IIB and IIC.	
